# CGCNImp: a causal graph convolutional network for multivariate time series imputation

**DOI:** 10.7717/peerj-cs.966

**Published:** 2022-04-29

**Authors:** Caizheng Liu, Guangfan Cui, Shenghua Liu

**Affiliations:** 1 Department of Data Science, Institute of Computing Technology, Chinese Academy of Sciences, Beijing, China; 2 Department of Computer Science and Technology, University of Chinese Academy of Sciences, Beijing, China

**Keywords:** Multivariate time series imputation, Graph causal analysis, Graph neural network, Deep neural network

## Abstract

**Background:**

Multivariate time series data generally contains missing values, which can be an obstacle to subsequent analysis and may compromise downstream applications. One challenge in this endeavor is the presence of the missing values brought about by sensor failure and transmission packet loss. Imputation is the usual remedy in such circumstances. However, in some multivariate time series data, the complex correlation and temporal dependencies, coupled with the non-stationarity of the data, make imputation difficult.

**Mehods:**

To address this problem, we propose a novel model for multivariate time series imputation called CGCNImp that considers both correlation and temporal dependency modeling. The correlation dependency module leverages neural Granger causality and a GCN to capture the correlation dependencies among different attributes of the time series data, while the temporal dependency module relies on an attention-driven long short term memory (LSTM) and a time lag matrix to learn its dependencies. Missing values and noise are addressed with total variation reconstruction.

**Results:**

We conduct thorough empirical analyses on two real-world datasets. Imputation results show that CGCNImp achieves state-of-the-art performance when compared to previous methods.

## Introduction

Multivariate time series data is common to many systems and domains–any data that changes value over time can be most naturally represented as a time series. This includes data captured by a sensor or measured at intervals, for example, traffic monitoring data (Wang et al., 2017; Zhang, Zheng & Qi, 2017), healthcare and patient monitoring data (Che et al., 2018; Suo et al., 2019; Liu & Hauskrecht, 2016), Industrial Internet of Things (IIoT) systems data, financial marketing data (Bauer, Schölkopf & Peters, 2016; Batres-Estrada, 2015) and so on. In these domains, data is typically extracted in the form of multivariate time series data. What is also common is missing values and noise brought about by sensor failure, transmission packet loss, human error, and other issues. Missing values will not only destroy the integrity and balance of original data distributions, but also affect the subsequent analysis and application of related scenarios (Cheema, 2014; Berglund et al., 2015). The processing of missing values in time series has become a very important problem. Some researches try to directly model the dataset with missing values (Zheng et al., 2017). However, for every dataset, we need to model them separately. In most cases, imputation of the missing values is the standard remedy, but imputing with multivariate time series data is not so easy. The complex correlation and temporal dependencies found in some multivariate time series data complicates matters, and the non-stationarity of the data only exacerbates the issue:

**Figure 1:**
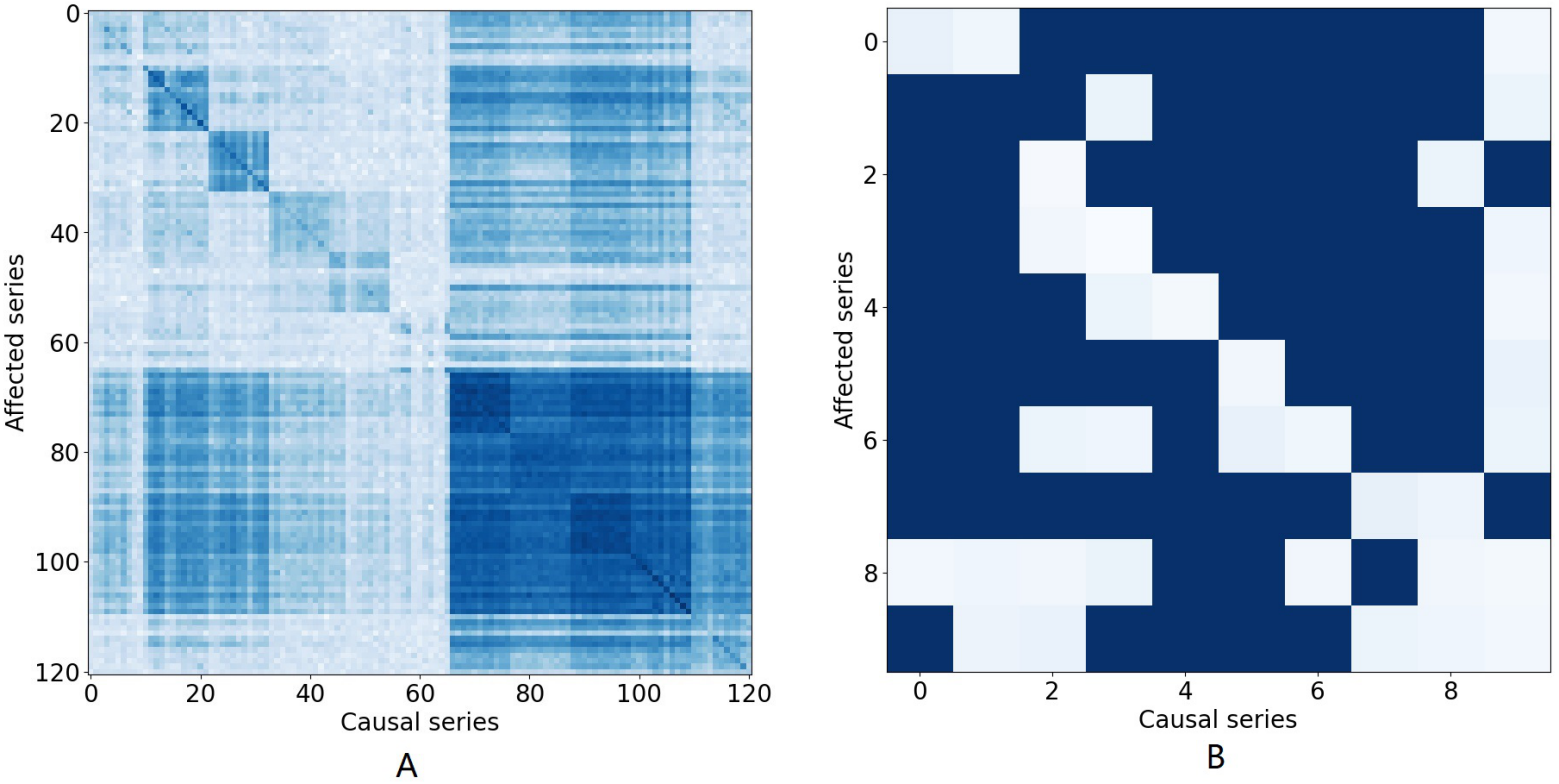
The causal effect matrix of KDD dataset and bird migration dataset. The *X* axis indicates attributes. The *Y* axis also indicates attributes. The matrix indicates the causal effect between attributes.

**Attribute correlation dependencies:** In many multivariate time series, it is important to interpret the attribute correlations within the time series that naturally arise. Typically, this correlation provides information about the contemporaneous and lagged relationships within and between individual series and how these series interact (Tank et al., 2021; Tank et al., 2018). Figure 1A illustrates the causal relationship graph with the KDD^1^ 1KDD CUP. Available on: http://www.kdd.org/kdd2018/, 2018.time series which collects air quality and weather data. In this data, there are 121 variables in total consisting of 11 different locations, each with 11 different variables. Different attributes for the same places are arranged in adjacent positions. A dark blue element (*i*, *j*) means that there is a strong Granger causal effect from variable *i* to variable *j*. It can be seen that the causal effect is strong along the diagonal of the matrix, which means that there are strong causal effects among different variables at the same location. Similarly, Fig. 1B shows causal effects between variables in a bird migration dataset that we study in this paper. Several research teams have also demonstrated that many aspects of weather, including temperature, precipitation, air pressure, wind speed, and wind direction, have substantial impacts on the migration of birds and that those impacts are inherently nonlinear (Clairbaux et al., 2019; Bozó, Csörgö & Heim, 2018). Hence, when attempting to impute missing values, all of these factors must be taken into account and the correlations between all these factors needs to be properly modeled to arrive at an accurate result.

**Temporal auto-correlation dependencies:** The evolution of multivariate time series changes dynamically over time and is mainly reflected in auto-correlations and trends (Anghinoni et al., 2021). For example, in the bird migration case, factors affecting these correlations can include inadequate food and subsequent starvation, too little energy to travel, bad weather conditions, and others Visser et al. (2009).

Researchers have proposed various methods of imputing missing values for time series data. The most recent techniques include using the complete data of existing observations to build a model or learn the data distribution and then using that distribution to estimate the missing values. The current models and algorithms with good prediction performance include imputation methods based on recurrent neural networks (RNNs) (Che et al., 2018; Suo et al., 2019) and generative adversarial networks (GANs) (Goodfellow et al., 2014). Recently, autoencoders have also been used to impute missing values in multivariate time series data. These represent the current state-of-the-art. For instance, Fortuin et al. (2020) proposed a model based on a deep autoencoder that maps the missing values of multivariate time series data into a continuous low-dimensional hidden space. This framework treats the low-dimensional representations as a Gaussian process but does not specify the goal of learning as the generation of real samples. Rather, the model simply tries to generate data that is close to a real sample. The result is a set of fuzzy samples. GlowImp (Liu et al., 2022) combines Glow-VAEs and GANs into a generative model that simultaneously learns to encode, generate and compare dataset samples. Although all these systems perform well at their intended task, none consider complex attribute correlations or temporal auto-correlation dependencies.

To fill this gap in the literature, we propose a novel model for multivariate time series imputation called CGCNImp that tackles two challenges. CGCNImp leverages neural Granger causality and a GCN to capture correlation dependencies between the attributes and an attention-driven LSTM plus a time lag matrix to model temporal auto-correlation dependencies and to impute the missing values. Last, neighbors with similar values are used to smooth the time series and reduce noise. In summary, our main contributions include:

• A novel model for imputing multivariate time series that considers both attribute correlation and temporal auto-correlation dependencies. The combination of neural Granger causality, an attention mechanism, and time lag decay yields satisfactory performance compared to the current methods.
• An imputation technique based on Granger causality and a GCN that captures attribute correlations for higher accuracy. In addition, an attention mechanism and total variation reconstruction automatically recovers latent temporal information.
• We conduct thorough empirical analyses on two real-world datasets. Imputation results show that CGCNImp achieves state-of-the-art performance when compared to previous methods.

Reproducibility: Our open-sourced code and the data used in this article are available at https://github.com/zhewen166/CGCNImp.

## Related Work

In recent years, researchers have generated a large body of literature on the imputation of missing values. Due to the limited space, we only describe a few closely related methods.

### Statistical methods

Statistical (Little & Rubin, 2019) imputation algorithms impute the missing values with the mean value (Kantardzic, 2011), the median value (na Edgar & Caroline, 2004), the mode value (Donders et al., 2006) or the last observed valid value (Amiri & Jensen, 2016).

### Machine learning based methods

Some researchers impute the missing values with machine learning algorithms showing that machine learning based imputation methods are useful for time series imputation. K-Nearest Neighbor (KNN) (Liew, Law & Yan, 2011) uses pairwise information between the target with missing values and the k nearest references to impute the missing values. Expectation- Maximization (EM) (Nelwamondo, Mohamed & Marwala, 2007) carries out a multi-step process which predicts the value of the current state and then applies to two estimators refining the predicted values of the given state, maximizing a likelihood function. The Matrix Factorization (MF) (Li et al., 2015) uses a low rank matrix to estimate the missing value. Tensor Singular Value Decomposition (t-SVD) (He, Guiling Sun & Geng, 2016) initializes the missing values as zeroes. It carries out an the SVD decomposition and selects the k most significant columns of V, using a linear combination of these columns to estimate the missing values. Multivariate Imputation by Chained Equations (MICE) (Azur et al., 2011; Buuren & Groothuis-Oudshoorn, 2011) uses a chained equation to fill the missing values. Autoregressive (Sridevi et al., 2011) modeling estimates missing values using an autoregressive model. The vector autoregressive imputation method (VAR-IM) (Bashir & Wei, 2018) is based on a vector autoregressive (VAR) model by combining an expectation and minimization algorithm with the prediction error minimization method. The Gradient-boosted tree (Friedman, 2020) model is built in a stage-wise fashion as in other boosting methods, but it generalizes the other methods by allowing optimization of an arbitrary differentiable loss function.

### Deep learning based methods

In time series imputation, deep learning based approaches can be classified into RNN-based methods, VAE-based methods, and GAN-based methods.

**RNN-Based methods.** GRU-D (Che et al., 2018) predicts the missing variable by the combination of last observed value, the global mean, and the time lag. But, it has drawbacks on general datasets (Che et al., 2018). M-RNN (Yoon, Zame & van der Schaar, 2017) utilizes a bi-directional RNN to impute missing values since both previous series and next series of missing values may be known in the scenario considered in their work. BRITS (Cao et al., 2018) uses the RNN structure to model the time series including the Unidirectional Uncorrelated Recurrent Imputation, Bidirectional Uncorrelated Recurrent Imputation , and Correlated Recurrent Imputation algorithms. All these models may suffer from the problem of vanishing or exploding gradients (Bengio et al., 2015) and error accumulation when encountering the continuous missing values.

**VAE-Based methods.** VAEs (Kingma & Welling, 2014) constitute a novel approach for efficient approximate inference with continuous latent variables. HI-VAE (Nazbal et al., 2020) deals with missing data on Heterogeneous and Incomplete Data. However, HI-VAE is not suitable for time series data as it does not exploit temporal information. GP-VAE (Fortuin et al., 2020) combines variational autoencoders and Gaussian processes for time series data. The VAE maps the missing data from the input space into a latent space where the temporal dynamics are modeled by the GP. GlowImp (Liu et al., 2022) combines Glow-VAEs and GANs into a generative model that simultaneously learns to encode, generate, and compare dataset samples. All these methods only optimize a lower bound and do not specify the goal of learning to generate real samples.

**GAN-Based methods.** Goodfellow et al. (2014) introduced the generative adversarial networks (GAN), and train generative deep models *via* an adversarial process. GAIN (Yoon, Jordon & van der Schaar, 2018) has some unique features. The generator receives random noise and a mask vector as input data and the discriminator gets some additional information *via* a hint vector to ensure that the generator generates samples depending on the true data distribution. But GAIN is not suitable for time series. GRUI-GAN (Luo et al., 2018) proposed a two second stage GAN based model. The generator tries to generate a realistic time series from the random noise vector z. The discriminator tries to distinguish whether the input data is real data or fake data. The adversarial structure can improve accuracy. However, this two-stage training needs a lot more time to train the best matched data and seems unstable with a random noise input (Luo et al., 2019). E2GAN (Luo et al., 2019) can impute the incomplete time series *via* an end-to-end strategy. This work proposes an encoder–decoder GRUI based structure as the generator, which can improve the accuracy and stability when training the model. The discriminator consists of a GRUI layer and a fully connected layer working as the encoder. SSGAN (Miao et al., 2021) is a novel semi-supervised generative adversarial network model, with a generator, a discriminator, and a classifier to predict missing values in the partially labeled time series data (Che et al., 2018; Cao et al., 2018; Luo et al., 2019).

## Methodology

### Motivation

In many multivariate time series, it is important to interpret the attribute correlations that naturally arise. Generally, these correlation can be divided into attribute correlation dependencies and temporal auto-correlation dependencies. Hence, our work includes three main considerations: these two types of dependencies plus end-to-end multi-task modeling to properly capture both.

**Attribute correlation dependency**. Typically, this correlation provides information about the contemporaneous and lagged relationships within and between individual series and how these series interact (Tank et al., 2021; Tank et al., 2018). For example, in the bird migration case, the main attribute dependencies are weather factors such as temperature, air pressure, and wind conditions. All can have a substantial impact on evolution of multivariate time series (Clairbaux et al., 2019; Bozó, Csörgö & Heim, 2018). These, therefore, need to be considered if one is to accurately impute any missing values. At the same time, there may be false correlation between some attributes. Hence, determining reasonable causal effects among different attributes is also an important issue. We opted for neural Granger causality (Tank et al., 2021; Tank et al., 2018) to model the correlation dependencies between the variables because it has achieved satisfactory performance on multivariate time series causal inferences, and it could be easily integrated into the multivariate time series imputation framework.

**Temporal auto-correlation dependency**. The evolution of multivariate time series changes dynamically over time and patterns are quasi-periodical on different scales of years and days (Anghinoni et al., 2021). Additionally, sensor malfunctions and failures, transmission errors, and other factors can mean the recorded time series carries noise (Han & Wang, 2013). Effectively exploiting auto-correlation relationships and eliminating sensor noise is therefore a key consideration.

**Multitask modeling**. Classical time series imputation methods adopt a two-stage modeling approach (Luo et al., 2018; Yoon, Jordon & van der Schaar, 2018; Miao et al., 2021). First, they analyze the correlations between multiple sequences and then impute the different sequences separately. However, these two-stage methods can not guarantee the global optimum. In this paper, we aim to establish an end-to-end model for Granger causal analysis and deep-learning-based time series imputation under the same framework, which will hopefully accelerate the imputation process and provide interpretability.

### Preliminary

**Definition 1: Multivariate Time Series.** A multivariate time series *X* = {*x*_1_, *x*_2_, …, *x*_*n*_} is a sequence with data observed at *n* timestamps *T* = (*t*_0_, *t*_1_, …, *t*_*n*−1_). The *i* − *th* observation *x*_*i*_ contains *d* attributes $({x}_{i}^{1},{x}_{i}^{2})$, …, ${x}_{i}^{d}$).

**Example 1: Multivariate Time Series.** We give an example of the multivariate time series *X* with missing values, / indicates the missing value.

\begin{eqnarray*}X= \left[ \begin{array}{@{}ccccc@{}} \displaystyle 5&\displaystyle /&\displaystyle /&\displaystyle /&\displaystyle 18\\ \displaystyle 12&\displaystyle 32&\displaystyle 9&\displaystyle /&\displaystyle 76\\ \displaystyle 2&\displaystyle /&\displaystyle 24&\displaystyle /&\displaystyle 47\\ \displaystyle \end{array} \right] \end{eqnarray*}

**Definition 2: Binary Mask Matrix.** Time series *X* may contain missing values, and a binary mask vector ℝ^*n*×*d*^ is introduced to indicate the missing positions, which is defined as:

\begin{eqnarray*}{M}_{i}^{j}= \left\{ \begin{array}{@{}ll@{}} \displaystyle 0, &\displaystyle \text{if}{x}_{i}^{j}\text{is null}\\ \displaystyle 1, &\displaystyle \text{otherwise}\\ \displaystyle \end{array} \right. \end{eqnarray*}

if the *j*th attribute of *x*_*i*_ is observed, ${M}_{i}^{j}$ is set to 1. Otherwise, ${M}_{i}^{j}$ is set to 0.

**Example 2: Binary Mask Matrix.** We can thus compute the binary mask matrix according to the multivariate time series X in example 1 which have missing values.

\begin{eqnarray*}M= \left[ \begin{array}{@{}ccccc@{}} \displaystyle 1&\displaystyle 0&\displaystyle 0&\displaystyle 0&\displaystyle 1\\ \displaystyle 1&\displaystyle 1&\displaystyle 1&\displaystyle 0&\displaystyle 1\\ \displaystyle 1&\displaystyle 0&\displaystyle 1&\displaystyle 0&\displaystyle 1 \end{array} \right] \end{eqnarray*}

**Definition 3: Time Lag Matrix.** In order to record the time lag between current value and last observed value, we introduce the time lag matrix *δ* ∈ *R*^*n*∗*d*^. The following formation shows the calculation of the *δ* from the last observation to the current timestamp *s*_*t*_.

\begin{eqnarray*}{\delta }_{t}^{d}= \left\{ \begin{array}{@{}ll@{}} \displaystyle {s}_{t}-{s}_{t-1}+{\delta }_{t-1}^{d} &\displaystyle \text{if}t\gt 0\text{and}{M}_{t-1}^{d}==0\\ \displaystyle {s}_{t}-{s}_{t-1} &\displaystyle \text{if}t\gt 0\text{and}{M}_{t-1}^{d}==1\\ \displaystyle 0 &\displaystyle \text{if t == 0} \end{array} \right. \end{eqnarray*}

### CGCNImp model

**Figure 2:**
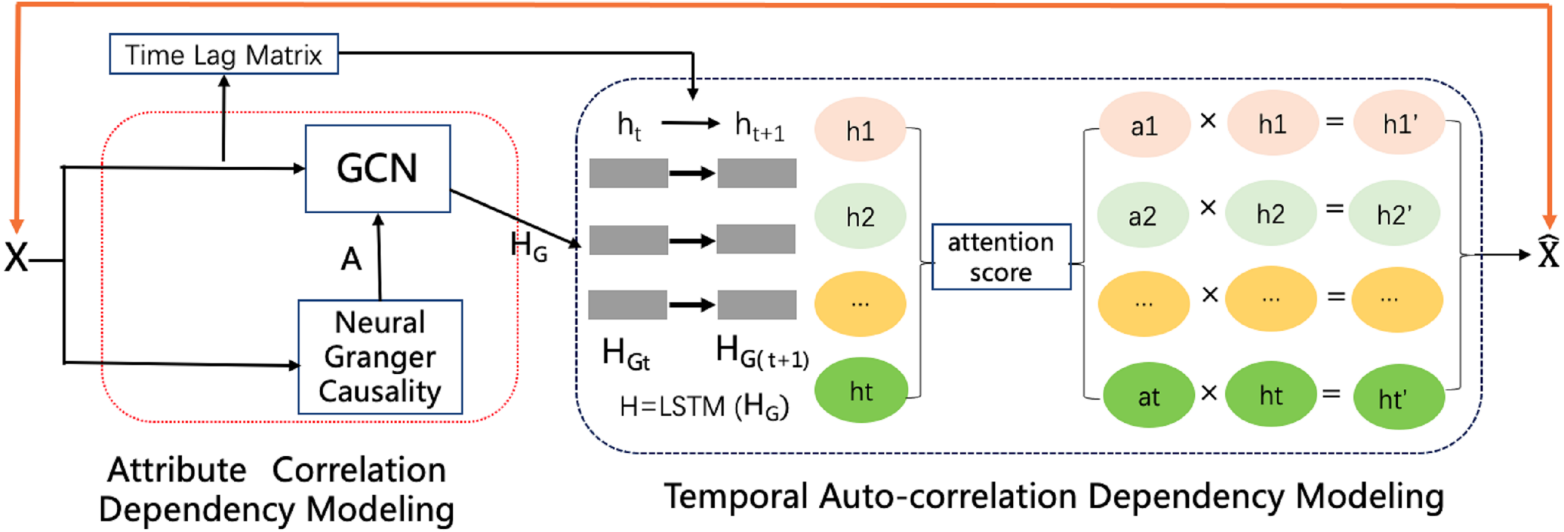
The CGCNImp framework for multivariate time series missing value imputing.

To impute reasonable values in place of the missing values, as shown in Fig. 2, the model contains an attribute correlation dependency module and a temporal auto-correlation dependency module. The correlation dependency module leverages neural Granger causality and a GCN to capture the correlation dependencies between attributes. The output of this module is passed to the temporal dependency module, which combines an attention-driven LSTM with a time lag matrix to impute the missing values. Last, a noise reduction and smoothness module uses neighbors with similar values to smooth the time series and remove much of the noise, while still preserving occasional rapid variations in the original signal. The details of each of these modules and the framework as a whole are discussed in the following sections.

#### Attributes causality modeling

Determining complex correlation dependencies is a key problem in the process of imputing with multivariate time series data. Here we use the neural Granger causality (Tank et al., 2021; Tank et al., 2018) to model the correlation dependency between the attributes of the multivariate time series. Let *x*_*t*_ ∈ *R*^*d*^ be a d-dimensional stationary time series and assume we have observed the process at n timestamps *T* = (*t*_0_, *t*_1_, …, *t*_*n*−1_). The basic idea of neural Granger causality is to gauge the extent to which the past activity of one time series is predictive of another time series. Thus, let *h*_*t*_ ∈ *R*^*d*^ represents the d-dimensional hidden state at time *t*, which represents the historical context of the time series for predicting a component *x*_*ti*_. The hidden state at time *t* + 1 is updated recursively

(1) \begin{eqnarray*}{h}_{t+1}=f({x}_{t},{h}_{t})\end{eqnarray*}

where *f* is some nonlinear function that depends on the particular recurrent architecture. We opted for an LSTM to model the recurrent function *f* due to its effectiveness at modeling complex time dependencies. The standard LSTM model takes the form:

(2) \begin{eqnarray*}{f}_{t}=\sigma ({W}_{f}{x}_{t}+{U}_{f}{h}_{t-1}){i}_{t}=\sigma ({W}_{i}{x}_{t}+{U}_{i}{h}_{t-1}){o}_{t}=\sigma ({W}_{o}{x}_{t}+{U}_{o}{h}_{(t-1)}){c}_{t}={f}_{t}\odot {c}_{t-1}+{i}_{t}\odot tanh({W}_{c}{x}_{t}+{U}_{c}{h}_{t-1}){h}_{t}={o}_{t}\odot tanh({c}_{t})\end{eqnarray*}

where ⊙ denotes component-wise multiplication and *i*_*t*_, *f*_*t*_, and *o*_*t*_ represent input, forget and output gates, respectively. These control how each component of the state cell *c*_*t*_, is updated and then transferred to the hidden state used for prediction *h*_*t*_.*W*_*f*_, *W*_*i*_, *W*_*o*_, *W*_*c*_, *U*_*f*_, *U*_*i*_, *U*_*o*_, *U*_*c*_ are the parameters that need to learn by LSTM. The output for series *i* is given by a linear decoding of the hidden state at time *t*:

(3) \begin{eqnarray*}{x}_{ti}={g}_{i}({x}_{\lt t})+{e}_{ti}\end{eqnarray*}

where the dependency of *g*_*i*_ on the full past sequence *x*_<*t*_ is due to recursive updates of the hidden state. The LSTM model introduces a second hidden state variable *c*_*t*_, referred to as the cell state, giving the full set of hidden parameter as (*c*_*t*_, *h*_*t*_).

In Eq. (2) the set of input maxtrices,

(4) \begin{eqnarray*}W=(({W}_{f})^{T},({W}_{i})^{T},({W}_{o})^{T},({W}_{c})^{T})^{T}\end{eqnarray*}

controls how the past time series *x*_*t*_, influences the forget gates, input gates, output gates, and cell updates, and, consequently, the update of the hidden representation. A group lasso penalty across the columns of *W* can be selected to indicate which Granger series causes series *i* during estimation. The loss function for modeling the attribute correlation dependencies is as follows:

(5) \begin{eqnarray*}{L}_{NG}={\min }_{W,U,{W}_{o}}\sum _{t=2}^{T}({x}_{it}-{g}_{i}({x}_{\lt t}))^{2}+\lambda \sum _{j=1}^{d}{|}{|}W{|}{{|}}_{1}\end{eqnarray*}

where *U* = (((*U*_*f*_)^*T*^, (*U*_*i*_)^*T*^, (*U*_*o*_)^*T*^, (*U*_*c*_)^*T*^)^*T*^) . The adjacent matrix *A*, which is produced by neural Granger causality, is stated as:

(6) \begin{eqnarray*}{A}_{ij}={|}{|}{W}_{{g}_{j}}^{i}{|}{\mathop{{|}\nolimits }\nolimits }_{F}^{2}\end{eqnarray*}

This adjacency matrix is used in the graph convolution network component discussed in the next subsection.

#### Attributes correlation dependency modeling

Convolutional neural networks (CNNs) can derive local correlation features but can only be used in Euclidean space. GCNs, however, are semi-supervised models that can handle arbitrary graph-structured data. As such, they have received widespread attention. GCNs can include spectrum and/or spatial domain convolutions. In this study, we use spectrum domain convolutions. In the Fourier domain, spectral convolutions on graphs are defined as the multiplication of a signal *x* with a filter *g*_*θ*_: *g*_*θ*_ ∗ *x* = *U g*_*θ*_(*U*^*T*^ *x*). Here *U* is the matrix of eigenvectors of the normalized graph Laplacian *L* = *I*_*N*_ − ${D}^{- \frac{1}{2} }A{D}^{- \frac{1}{2} }=U$ *λ U*^*T*^, *U*^*T*^*x* is the graph Fourier transform of *x*, *A* ∈ *R*^*d*∗*d*^ is an adjacency matrix and *λ* is diagonal matrix of its eigenvalues. In multivariate time series, *x* can also be a *X* ∈ *R*^*n*∗*d*^, where *d* refers to the number of features and *n* refers to the time internals. Given the adjacent matrix *A* which is produced by neural Granger causality, GCNs can perform the spectrum convolutional operation to capture the correlation characteristics. The GCN model can be expressed as:

(7) \begin{eqnarray*}{H}_{G}=\sigma ({\widetilde {W}}^{- \frac{1}{2} }\widetilde {A}{\widetilde {W}}^{- \frac{1}{2} }X\theta )\end{eqnarray*}

where $\widetilde {A}=A+{I}_{N}$ is an adjacent matrix with self-connection structures, *I*_*N*_ is an identity matrix, $\widetilde {W}$ is a degree matrix, *H*_*G*_ ∈ *R*^*n*∗*d*^ is the output of GCN which is the input of the temporal auto-correlation dependency modeling, *θ* is the parameter of GCN, and *σ*(⋅) is an activation function used for nonlinear modeling.

#### Temporal auto-correlation dependency modeling

Obtaining complex temporal auto-correlation dependencies is another key problem with imputation of multivariate time series data. In particular, sometimes the input decay may not fully capture the missing patterns since not all missingness information can be represented in decayed input values. Due to its effectiveness at modeling complex time dependencies, we choose to model the temporal dependencies using an LSTM (Graves, 2012). However, to properly learn the characteristics of the original incomplete time series dataset, we find that the time lag between two consecutive valid observations is always changing due to the nil values. Further, the time lags between observations are very important since they follow an unknown non-uniform distribution. These changeable time lags remind us that the influence of the past observations should decay with time if a variable has been missing for a while.

Thus, a time decay vector *α* is introduced to control the influence of the past observations. Each value of *α* should be greater than 0 and smaller than 1 with the larger the *δ*, the smaller the decay vector. Hence, the time decay vector *α* is modeled as a combination of *δ*:

(8) \begin{eqnarray*}{\alpha }_{t}=1/{e}^{max(0,{W}_{\alpha }{\delta }_{t}+{b}_{\alpha })}\end{eqnarray*}

where *W*_*α*_ and *b*_*α*_ are parameters that need to be learned. Once the decay vector has been derived, the hidden state in the LSTM *h*_*t*−1_ is updated in an element-wise manner by multiplying the decay vector *α*_*t*_ to fit the decayed influence of the past observations. Thus, the update functions of the LSTM are as follows:

(9) \begin{eqnarray*}{h}_{t-1}^{{}^{{^{\prime}}}}={\alpha }_{t}\odot {h}_{t-1}{i}_{t}=\sigma ({W}_{i}[{h}_{t-1}^{{}^{{^{\prime}}}};{H}_{{G}_{t}}]+{b}_{i}){f}_{t}=\sigma ({W}_{f}[{h}_{t-1}^{{}^{{^{\prime}}}};{H}_{{G}_{t}}]+{b}_{f}){s}_{t}={f}_{t}\odot {s}_{t-1}+{i}_{t}\odot tanh({W}_{s}[{h}_{t-1}^{{}^{{^{\prime}}}};{H}_{{G}_{t}}]+{b}_{s}){o}_{t}=\sigma ({W}_{o}[{h}_{t-1}^{{}^{{^{\prime}}}};{H}_{{G}_{t}}]+{b}_{o}){h}_{t}={o}_{t}\odot tanh({s}_{t})\end{eqnarray*}

where *W*_*f*_, *W*_*i*_, *W*_*o*_, *W*_*c*_, *b*_*f*_, *b*_*i*_, *b*_*o*_, *b*_*s*_ are the parameters that need to be learned by the by LSTM and *H*_*G*_ is the output of the attribute correlation dependency modeling.

Attentive neural networks have recently demonstrated success in a wide range of tasks and, for this reason, we use one here. Let *H*_*L*_ be a matrix consisting of output vectors *H*_*L*_ = [*h*_1_, *h*_2_, …, *h*_*n*_] ∈ℝ^*T*×*d*^ that the LSTM layer produced, where n is the time series length. The representation *β*_*ij*_ of the attention score is formed by a weighted sum of these output vectors:

(10) \begin{eqnarray*}{\beta }_{ij}= \frac{exp(tanh(W[{h}_{i}{|}{h}_{j}]))}{\sum _{k=1}^{T}exp(tanh(W[{h}_{k}{|}{h}_{j}]))} \end{eqnarray*}

(11) \begin{eqnarray*}{H}^{{}^{{^{\prime}}}}=Atte{n}_{L}\times {H}_{L}\end{eqnarray*}

where the $Atte{n}_{L}= \left[ {\scriptsize \begin{array}{@{}c@{}} \displaystyle {\beta }_{11},{\beta }_{12},\ldots ,{\beta }_{1T}\\ \displaystyle {\beta }_{21},{\beta }_{22},\ldots ,{\beta }_{2T}\\ \displaystyle \cdots \\ \displaystyle {\beta }_{n1},{\beta }_{n2},\ldots ,{\beta }_{nT}\\ \displaystyle \end{array}} \right] $ is the attention score.

In Eq. (11), We reconstruct the missing value by some linear transformation of the hidden state *H*′ at time *t*. Hence the reconstruction loss is formulated as:

(12) \begin{eqnarray*}{L}_{reg}=\sum _{x\in D}{ \left\| x\otimes m-\hat {x}\otimes m \right\| }_{2}\end{eqnarray*}

x represents the input multivariate time series data, $\hat {x}$ represents the imputed multivariate time series data and m means the masking matrix. The expression in Eq. (12) is the masked reconstruction loss that calculates the squared errors between the original observed data x and the imputed sample. Here, it should be emphasized that when calculating the loss, we only calculate the observed data as previously described in Cao et al. (2018), Luo et al. (2018), Luo et al. (2019) and Liu et al. (2022).

#### Noise reduction and smoothness imputation

In the past, reconstructions were performed directly, which ignores the noise in the actual sampling process. However, in real-world multivariate time series data, when time series are collected the observations may be contaminated by various types of error or noise. Hence, these imputation values may be unreliable. To ensure the reliability of the imputation results, a total variation reconstruction regularization term is applied to the reconstruction results. The method is based on a smoothing function where neighbors with similar values are used to smooth the time series. When applied to time series data, abrupt changes in trend, spikes, dips and the like can all be fully preserved. Compared to a two-norm smoothing constraint, the total variation reconstruction term can ensure smoothness without losing the dynamic performance of the time series (Boyd & Vandenberghe, 2004). Equation (13) applies the total variation reconstruction term to the reconstruction results. As a result, noise in the original data is reduced and completion accuracy is improved. The reconstruction loss is formulated as:

(13) \begin{eqnarray*}{L}_{SL}=\sum _{j=1}^{M}\sum _{i=1}^{N}{|}{|}{\hat {x}}_{i+1}^{j}-{\hat {x}}_{i}^{j}{|}{{|}}_{1}\end{eqnarray*}

where *M* is the number of time series, that is, the number of variables, and *N* is the length of each time series.

The total object function of our model is:

(14) \begin{eqnarray*}{L}_{loss}=\alpha \ast {L}_{NG}+\beta \ast {L}_{reg}+\theta \ast {L}_{SL}\end{eqnarray*}

where *α*, *β*, *θ* indicate the weights among different parts of the total loss. We optimize Eq. (14) by Adam optimizer.

## Experiment

To verify and measure the performance of the proposed CGCNImp framework, we compare its performance at imputation with multiple time series against several other contemporary methods. The selected datasets used in the evaluations were three real-world bird migration datasets focusing on migratory patterns in China—Anser albifrons and Anser fabalis—as well as the KDD 2018 CUP Dataset.

### Dataset description

#### KDD CUP 2018 dataset

The KDD dataset comes from the KDD CUP Challenge 2018. The dataset, which is is a public meteorologic dataset, has about 15% missing values. It was collected hourly between 2017/1/20 to 2018/1/30 in Beijing, collecting air quality and weather data. Each record contains 12 attributes, for example CO, weather, temperature etc. In our experiment, we select 11 common features as the comparison methods described in the next section. We split this dataset for every hour. For every 48 h, we randomly drop *p* percent of the dataset, and then we impute these time series with different models and calculate the imputation accuracy by RMSE and MAE where *p* ∈ {10, 20, 30, 40, 50, 60, 70, 80, 90}.

#### Bird migration dataset in China

The Birds Migration Dataset collects migration trace data which comes from the project Strategic Priority Research Program of the Chinese Academy of Sciences. The dataset was collected hourly between 2017/12/30 to 2018/5/10 based on the species Anser fabalis and Anser albifrons. Each record contains 13 attributes which are longitude, latitude, speed height, speed velocity, heading, temperature etc. The dataset is about 10% missing values. We select 10 common features comprising longitude, latitude, speed height, speed velocity, heading, temperature etc. for our experiments. We split this dataset into 5 min time series, and for every 5 min, we randomly drop *p* percent of the dataset, and then we impute these time series with different models and calculate the imputation accuracy by RMSE and MAE between original values and imputed values where *p* ∈ {10, 20, 30, 40, 50, 60, 70, 80, 90}.

### Comparison methods and evaluation metrics

We compare our methods to eight current imputation methods as previously described in (Liu et al., 2022). A brief description of each follows.

• Statistical imputation methods (Rubinsteyn & Feldman, 2016), where we simply impute the missing values with zero, mean, or median.
• KNN (Liew, Law & Yan, 2011), which imputes the missing data as the weighted average of k neighbors by using a k-nearest neighbor algorithm to find neighboring data.
• MF (Li et al., 2015), which fills the missing values through factorizing an incomplete matrix into low-rank matrices.
• SVD (He, Guiling Sun & Geng, 2016), which uses iterative singular value decomposition for matrix imputation to impute the missing values.
• GP-VAE (Fortuin et al., 2020), a method that combines ideas from VAEs and Gaussian processes to capture temporal dynamics for time series imputation.
• BRITS (Cao et al., 2018), one of methods that include Unidirectional Uncorrelated Recurrent Imputation, Bidirectional Uncorrelated Recurrent Imputation, and Correlated Recurrent Imputation algorithm to impute the missing values.
• GRUI (Luo et al., 2018), which is a two-stage GAN based method that uses a generator and discriminator to impute missing values.
• E2E-GAN (Luo et al., 2019) which relies on an end-to-end GAN network that includes an encoder–decoder GRUI based structure and is one of the state-of-the-art methods.

To evaluate the performance of our methods, we use two metrics to the compare and analyze with the results of previous methods. (1) RMSE (Root Mean Squared Error) refers to the mean value of the square root of the error between the predicted value and the true value. The calculation formula is as follows:

\begin{eqnarray*}RMSE=\sqrt{ \frac{1}{n} \sum _{i=1}^{n}(x-\hat {x})^{2}}. \end{eqnarray*}

(2) MAE (Mean Absolute Error) is the average of the absolute value of the error between the observed value and the real value. It is used to describe the error between the predicted value and the real value. The formulation is as follows:

\begin{eqnarray*}MAE= \frac{1}{n} \sum _{i=1}^{n}{|}x-\hat {x}{|}. \end{eqnarray*}

### Implementations details

All the experimental results are obtained under the same hardware and software environment. The hardware is Intel i7 9700 k, 48 GB memory, NVIDIA GTX 1080 8 GB. And the deep learning framework is PyTorch1.7 and TensorFlow1.15.0.

To maintain the same experiment environment as the contemporary method, the dataset was split into two parts: the first part with 80% of the records is used for the training set and the remaining 20% is used for the test set. All values are normalized within the range of 0 to 1. For the training process, 10% of the data of the training set was randomly dropped. For the testing dataset, we drop the data with different drop-rate between 10% and 90%, thus testing each method at a range of levels of missing data between 10% and 90%.

### Performance analysis

The results with the KDD, Anser albifrons and Anser fabalis datasets at a missing value ratio of 10% appear in Table 1. Here, CGCNImp yields significantly lower errors than the other methods in terms of RMSE and MAE, demonstrating that our method is better than the other methods.

Generally, the higher the proportion of missing data, the more difficult it is to impute the missing values. To assess the frameworks with different levels of missing data, we then conduct the same experiment with the BRITS, GRUI, E2EGAN and CGCNImp, varying the ratios of missing values from 10% to 90% in steps of 10% as previously described in Liu et al. (2022). The results are shown in Tables 2 and 3. Again, our methods return the smallest errors.

**Figure 3:**
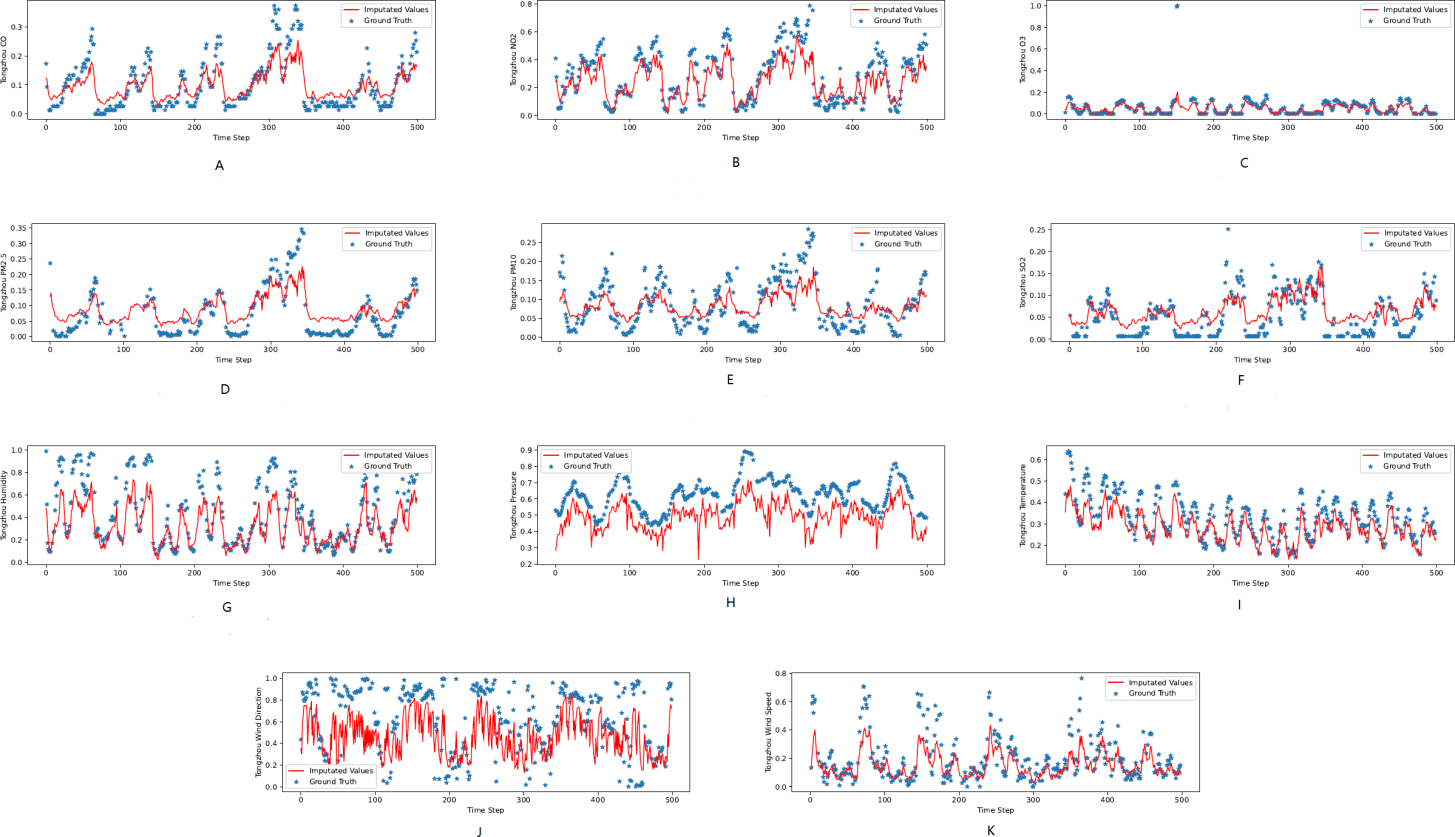
(A–K) The ground true (blue) and the imputed values (red) in Mentougou, Beijing of the KDD dataset. The *X* axis indicates time step. The *Y* axis indicates imputed values.

**Figure 4:**
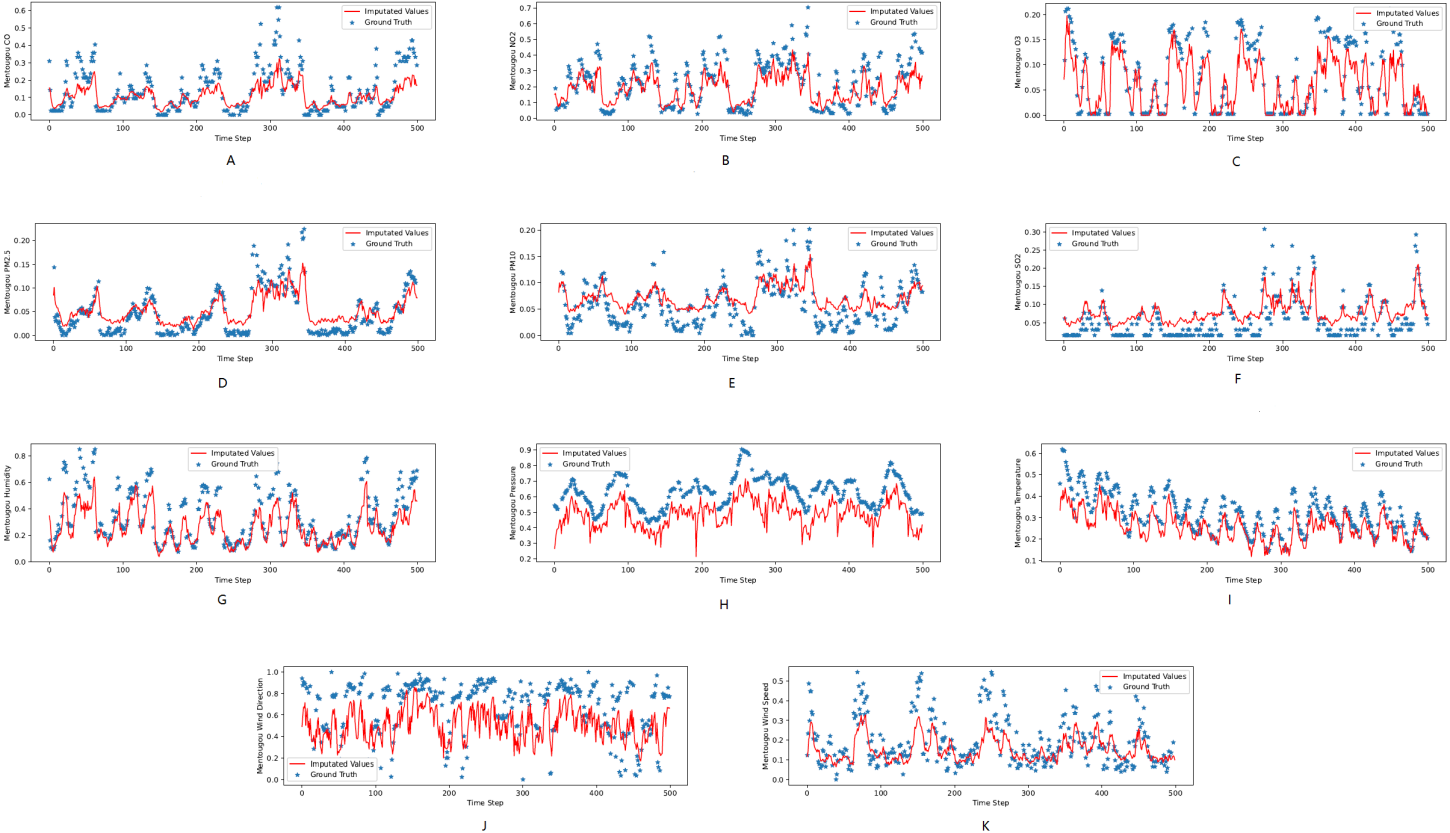
(A–K) The ground true (blue) and the imputed values (red) in Tongzhou, Beijing of the KDD dataset. The *X* axis indicates time step. The *Y* axis indicates imputed values.

**Figure 5:**
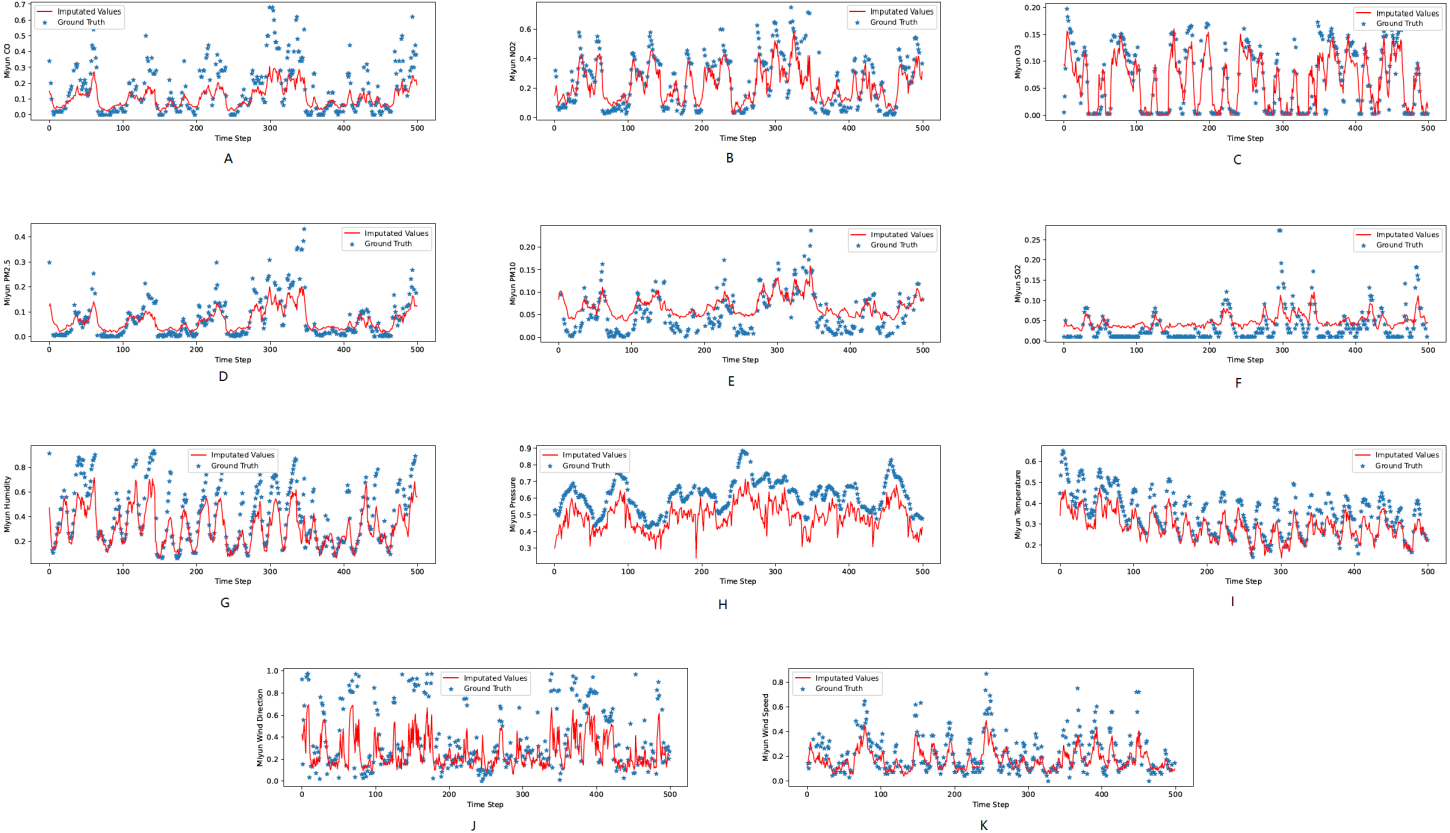
The ground true (blue) and the imputed values (red) in Miyun, Beijing of the KDD dataset. The *X* axis indicates time step. The *Y* axis indicates imputed values.

Figures 3, 4 and 5 show the imputation results from the KDD datasets for the Tongzhou, Mentougou and Miyun districts, respectively. The blue dots are the ground truth time series and the red curve shows the imputed values. As illustrated, CGCNImp captures the evolution of the values and imputes the missing values quite well.

**Table 1:** The RMSE and MAE results of the CGCNImp and other methods on the three datasets (lower is better).

Dataset	KDD dataset		Anser albifrons dataset		Anser fabalis dataset	
Method	RMSE	MAE	RMSE	MAE	RMSE	MAE
Zero	1.081	1.041	1.088	1.047	1.089	1.054
Mean	1.063	1.035	1.033	1.025	1.043	1.035
Random	1.821	1.637	1.802	1.431	1.721	1.677
Median	1.009	0.994	1.109	1.042	1.001	0.998
KNN	0.803	0.724	0.758	0.714	0.824	0.817
MF	0.784	0.627	0.643	0.626	0.663	0.646
SVD	1.043	0.966	1.253	1.051	1.129	1.011
GP-VAE	0.597	0.486	0.693	0.572	0.534	0.375
BRITS	0.156	0.148	0.159	0.124	0.137	0.078
GRUI	0.149	0.102	0.152	0.113	0.138	0.086
E2E-GAN	0.133	0.074	0.139	0.081	0.116	0.066
Ours	**0.114**	**0.062**	**0.128**	**0.072**	**0.107**	**0.059**

**Notes.**

Experimental results are shown in bold.

**Table 2:** The MAE results of the imputation methods on the three datasets with different missing rate (lower is better).

		Missing rate (%)								
		10	20	30	40	50	60	70	80	90
	BRITS	0.1484	0.1587	0.1722	0.1924	0.2076	0.2391	0.2624	0.3076	0.3591
	GRUI	0.1026	0.1397	0.1522	0.1684	0.1876	0.1971	0.2341	0.2863	0.3198
KDD	E2EGAN	0.0747	0.1087	0.1292	0.1428	0.1576	0.1796	0.1981	0.2076	0.2590
	Ours	**0.0624**	**0.0721**	**0.0814**	**0.0959**	**0.1109**	**0.1238**	**0.1397**	**0.1564**	**0.1745**
	BRITS	0.1242	0.1289	0.1331	0.1446	0.1576	0.1891	0.2021	0.2386	0.2896
	GRUI	0.1133	0.1197	0.1202	0.1377	0.1478	0.1691	0.1723	0.1976	0.2259
albifrons	E2EGAN	0.0815	0.0967	0.1026	0.1124	0.1389	0.1582	0.1648	0.1870	0.1993
	Ours	**0.0721**	**0.0785**	**0.0923**	**0.0992**	**0.1152**	**0.1346**	**0.1537**	**0.1714**	**0.2014**
	BRITS	0.0782	0.1371	0.1462	0.1639	0.1978	0.2123	0.2694	0.2971	0.3791
	GRUI	0.0863	0.1007	0.1252	0.1504	0.1771	0.1925	0.2492	0.3084	0.3271
fabalis	E2EGAN	0.0661	0.0787	0.0928	0.1027	0.1285	0.1382	0.1537	0.2004	0.2596
	Ours	**0.0591**	**0.0688**	**0.0815**	**0.0928**	**0.1099**	**0.1257**	**0.1467**	**0.1776**	**0.2189**

**Notes.**

Experimental results are shown in bold.

### Ablation study

An ablation study is designed to assess the contribution of the attribute causality discovery and the noise reduction and smoothness imputation. This comprised three tests: the first with no ablation; the second where we simply removed the noise reduction and smoothness module and set *β* to 0 in Eq. (14); plus a third where we simply removed the noise reduction and smoothness module and set *α* to 0 in in Eq. (14). All tests are conducted with a range of missing value ratios. Tables 4 and 5 show the results. What we found with the Anser bird migration data was that, at a missing rate lower than 40%, removing either the noise reduction and smoothness module or the neural Granger causality gives lower errors. However, at higher missing rates, the tests with both modules returned substantially lower errors. This verifies the contribution of both modules to the framework. With the KDD data, CGCNImp in full returned substantially lower errors, again supporting the contribution of both these modules.

**Table 3:** The RMSE results of the imputation methods on the three datasets with different missing rate (lower is better).

		Missing rate (%)								
		10	20	30	40	50	60	70	80	90
	BRITS	0.1561	0.1721	0.1928	0.2120	0.2571	0.2980	0.3284	0.3625	0.3912
KDD	GRUI	0.1493	0.1527	0.1702	0.1937	0.2098	0.2541	0.2824	0.3051	0.3361
	E2EGAN	0.1336	0.1457	0.1601	0.1778	0.1926	0.2235	0.2574	0.2808	0.3031
	Ours	**0.1142**	**0.1279**	**0.1402**	**0.1610**	**0.1803**	**0.2026**	**0.2263**	**0.2509**	**0.2776**
	BRITS	0.1596	0.1706	0.1931	0.2126	0.2398	0.2571	0.2964	0.3351	0.3686
albifrons	GRUI	0.1394	0.1562	0.1799	0.1971	0.2205	0.2483	0.2670	0.2995	0.3297
	E2EGAN	0.1289	0.1358	0.1572	0.1704	0.1976	0.2371	0.2480	0.2746	0.3098
	Ours	**0.1287**	**0.1394**	**0.1589**	**0.1679**	**0.1902**	**0.2163**	**0.2389**	**0.2590**	**0.2921**
	BRITS	0.1372	0.1451	0.1680	0.1901	0.2273	0.2398	0.2647	0.3004	0.3469
fabalis	GRUI	0.1381	0.1483	0.1761	0.2007	0.2492	0.2703	0.2906	0.3209	0.3501
	E2EGAN	0.1160	0.1246	0.1508	0.1688	0.1898	0.2103	0.2562	0.2953	0.3391
	Ours	**0.1076**	**0.1242**	**0.1444**	**0.1588**	**0.1829**	**0.2059**	**0.2323**	**0.2713**	**0.3205**

**Notes.**

Experimental results are shown in bold.

Figure 1A illustrates the causal relationship graph with the KDD time series. In this data, there are 121 variables in total, corresponding to 11 different locations, each with 11 different variables. Different attributes for the same places are arranged in adjacent positions. A dark blue element (*i*, *j*) means that there is a strong Granger causal effect from variable *i* to variable *j*. It can be seen that the causal effect is strong along the diagonal of the matrix, which means that there are strong causal effects among different variables at the same location. Furthermore, there are also strong causal effects between different locations, such as variables 66 to 110.

There are nine attributes in the bird migration dataset. Figure 1B shows the Granger causal matrix derived from the neural Granger causality analysis. It should be noted that the Granger causality should be a one-way relationship, which means that, theoretically, we need to eliminate conflicting edges in the causal graph. However, in practice, the causal graph is derived from the neural Granger causality analysis and the edge indicate there are strong correlations between variables. Therefore, we kept the conflicting edge and placed them into the GCN network for better performance.

### CASE STUDY: bird migration route analysis

**Figure 6:**
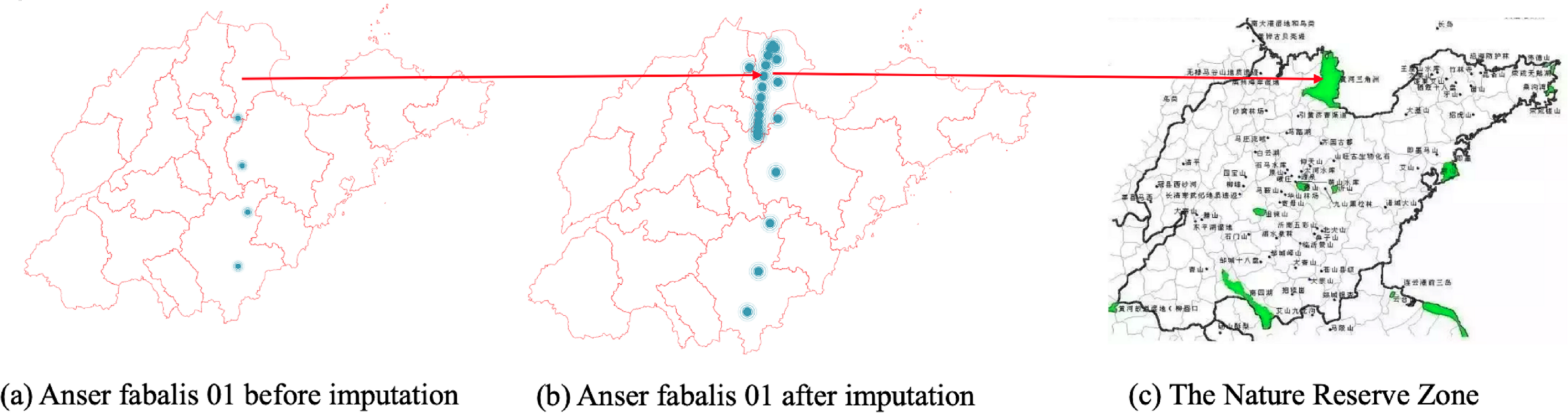
(A–C) Anser fabalis dataset imputation.

**Figure 7:**
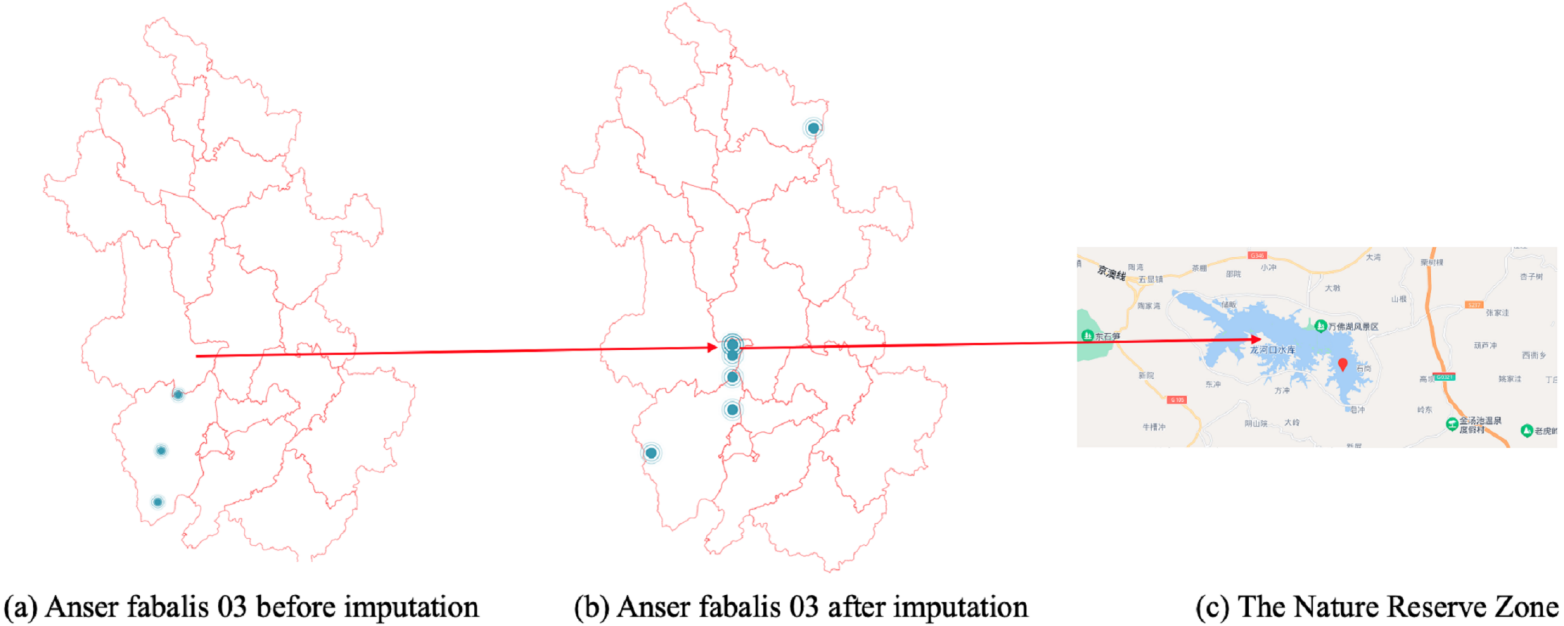
(A–C) Anser fabalis dataset imputation.

**Figure 8:**
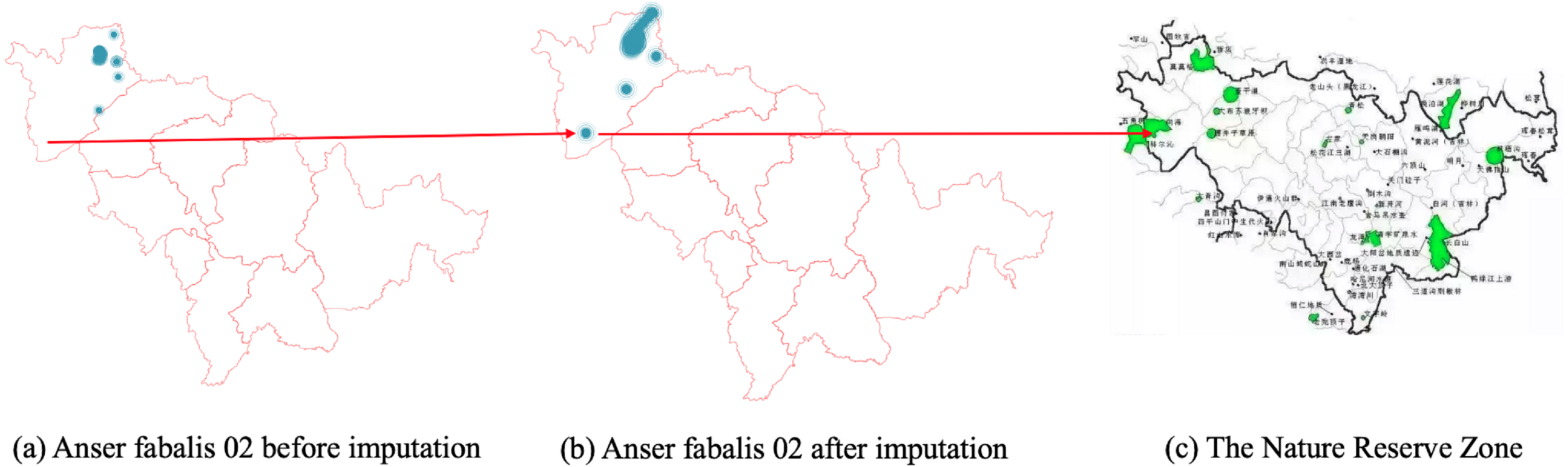
(A–C) Anser fabalis dataset imputation.

Figures 6, 7 and 8 show the imputation results of Anser fabalis birds migration routes. What we can see is that the imputed data shows some important wild reserves not seen with the original data. According to the list of wetlands of international importance in China, for example, Fig. 6B shows the ground truth time series with missing values. This time, CGCNImp imputed the location of Binzhou Seashell Island and the Wetland National Nature Reserve not shown in Fig. 6A showing that the bird migration trajectory could be recovered by our methods.

**Table 4:** The ablation study RMSE results of the CGCNImp method on the three datasets (lower is better).

		Missing rate (%)								
		10	20	30	40	50	60	70	80	90
	*θ* = 0	0.1468	0.1607	0.1713	0.1890	0.2071	0.2251	0.2449	0.2634	0.2845
KDD	*α* = 0	0.1278	0.1445	0.1607	0.1788	0.1982	0.2173	0.2372	0.2611	0.2826
	no ablation	**0.1142**	**0.1279**	**0.1402**	**0.1610**	**0.1803**	**0.2026**	**0.2263**	**0.2509**	**0.2776**
	*θ* = 0	0.1240	0.1329	0.1567	0.1726	0.1918	0.2143	0.2416	0.2694	0.3112
albifrons	*α* = 0	**0.1180**	**0.1301**	**0.1473**	0.1761	0.1873	0.2098	0.2304	0.2639	0.3047
	no ablation	0.1287	0.1394	0.1589	**0.1679**	**0.1802**	**0.2063**	**0.2289**	**0.2590**	**0.2921**
	*θ* = 0	0.1156	0.1296	**0.1373**	**0.1582**	0.1827	0.2092	0.2437	0.2833	0.3276
fabalis	*α* = 0	0.1169	0.1294	0.1393	0.1617	**0.1781**	0.2132	0.2359	0.2781	0.3230
	no ablation	**0.1076**	**0.1242**	0.1444	0.1588	0.1829	**0.2059**	**0.2323**	**0.2713**	**0.3205**

**Notes.**

Experimental results are shown in bold.

**Table 5:** The ablation study MAE results of the CGCNImp method on the three datasets (lower is better).

		Missing rate (%)								
		10	20	30	40	50	60	70	80	90
	*θ* = 0	0.0801	0.0925	0.1028	0.1155	0.1289	0.1416	0.1552	0.1688	0.1833
KDD	*α* = 00.0717	0.0835	0.0957	0.1090	0.1224	0.1356	0.1492	0.1658	0.1806	0.1866
	no ablation	**0.0624**	**0.0721**	**0.0814**	**0.0959**	**0.1109**	**0.1238**	**0.1397**	**0.1564**	**0.1745**
	*θ* = 0	0.0659	0.0739	0.0891	0.1012	0.1163	0.1327	0.1528	0.1764	0.2118
albifrons	*α* = 0	**0.0638**	**0.0722**	**0.0833**	0.1001	0.1112	0.1284	0.1457	0.1723	0.2083
	no ablation	0.0721	0.0785	0.0923	**0.0992**	**0.1052**	**0.1246**	**0.1437**	**0.1714**	**0.2014**
	*θ* = 0	0.0618	0.0721	0.0799	0.0935	0.1106	0.1298	0.1569	0.1879	0.2265
fabalis	*α* = 0	0.0633	0.0709	**0.0795**	0.0947	0.1079	0.1315	0.1497	0.1825	0.2218
	no ablation	**0.0591**	**0.0688**	0.0815	**0.0928**	**0.1069**	**0.1257**	**0.1467**	**0.1776**	**0.2189**

**Notes.**

Experimental results are shown in bold.

In Fig. 7B, the CGCNImp method imputed the location of Wanfoshan Nature Reserve, which is not noticeable in the original data on its own (Fig. 7A). Wanfoshan is now a national forest park, a national nature reserve, and a national geological park, which is an important location for bird migration.

Likewise, Fig. 8B shows the imputed location of the Momoge National Nature Reserve (Cui, Liu & S, 2021) not showed in Fig. 8A.

## Conclusion

In this paper, we present a novel imputation model, called CGCNImp, that is specifically designed to perform imputation of multivariate time series data. CGCNImp considers both attribute correlation and temporal auto-correlation dependencies. Correlation dependencies are captured through neural Granger causality and a GCN, while an attention-driven LSTM plus a time lag matrix capture the temporal dependencies and impute the missing values. Last, neighbors with similar values are used to smooth the time series and reduce noise. Imputation results show that CGCNImp achieves state-of-the-art performance when compared to previous methods. We will explore our model for missing-not-at-random data, and we will conduct a theoretical analysis of our model for missing values in further works.

## Supplemental Information

10.7717/peerj-cs.966/supp-1 Supplemental Information 1 Code

10.7717/peerj-cs.966/supp-2 Supplemental Information 2 Raw data

## Additional Information and Declarations

### Data Availability

The following information was supplied regarding data availability: The KDD CUP 2018 Dataset is available at GitHub: https://github.com/zhewen166/CGCNImp.
